# Layer‐specific strain echocardiography may reflect regional myocardial impairment in patients with hypertrophic cardiomyopathy

**DOI:** 10.1186/s12947-021-00244-3

**Published:** 2021-03-03

**Authors:** Zhongxiu Chen, Chunmei Li, Yajiao Li, Li Rao, Xiaoling Zhang, Dan Long, Chen Li

**Affiliations:** 1 Department of Cardiology, West China Hospital of Sichuan University, 37 Guo Xue Xiang, 610041 Chengdu, Sichuan China; 2 Key Laboratory of Transplant Engineering and Immunology, West China Hospital of Sichuan University, High-tech Zone, Chengdu, Sichuan China

**Keywords:** Layer‐specific strain, Hypertrophic cardiomyopathy, Myocardial impairment, Microvascular dysfunction

## Abstract

Our study aimed to determine whether layer-specific strain (LSS) could reflect regional myocardial impairment in patients with hypertrophic cardiomyopathy (HCM). The study enrolled 50 patients with HCM and 30 age-matched healthy controls. Transmural gradient of longitudinal strain (TGLS), defined as the difference between the longitudinal strain of the endocardium and epicardium in a left ventricular segment, was used to reflect layer-specific myocardial impairment. Negative TGLS was consistently observed in healthy controls. The TGLS was relatively consistent within the basal, middle, and apical levels in healthy controls,but showed a significant gradient from the base towards the apex. In patients with HCM, the hypertrophic segments had significantly higher TGLS than the relatively normal segments or healthy controls at all 3 levels (0.14 % ± 3.48 % vs. −2.65 % ± 4.44 % vs. −2.17 % ± 1.66 % for basal, − 0.72 % ± 3.71 % vs. −4.02 % ± 4.00 % vs. −3.58 % ± 2.29 % for middle, and − 8.69 % ± 7.96 % vs. −11.44 % ± 6.65 % vs. −10.04 % ± 3.20 % for apex). Abnormal TGLS, defined as positive TGLS, in patients with HCM was associated with chest pain. In receiver operating characteristic curve analysis, a large area of abnormal TGLS (> 4 segments) had moderate accuracy for predicting chest pain (sensitivity, 73.3 %; specificity, 70.0 %). TGLS, a novel LSS derived parameter, may reflect regional myocardial impairment in patients with HCM.

## Key messages

Patients with hypertrophic cardiomyopathy (HCM) could develop layer-specific myocardial impairment because of subendocardial microvascular dysfunction and focal cardiac fibrosis, however, this phenomenon has not received significant attention due to the lack of a feasible examination method.

The hypertrophic left ventricular segments had significantly higher TGLS, defined as the difference between territorial longitudinal strain of the endocardium and the epicardium, than the relatively normal segments of HCM patients or healthy controls.

Positive TGLS, which was consistently negative in healthy controls, was associated with chest pain in HCM patients, and may reflect endocardium-specific impairment of regional heart function in patients with HCM.

## Introduction

Two-dimensional (2-D) speckle-tracking echocardiography is widely acknowledged as a sensitive method for evaluating global and regional left ventricular (LV) function. The ventricular wall consists of 3 layers, namely, the epicardium, mid-myocardium, and endocardium. Conventionally, assessment of the ventricular wall has not taken into account the different layers of the myocardium but has considered them as a single functional unit. This simplification generally works well. However, in patients with hypertrophic cardiomyopathy (HCM), this could be problematic. Previous studies have demonstrated that patients with HCM could develop subendocardial microvascular dysfunction [1] and focal cardiac fibrosis [2, 3]. These abnormalities could potentially lead to layer-specific myocardial impairment. However, the possibility of layer-specific myocardial impairment has not received significant attention due to the lack of a feasible examination method.

Improvements in the temporal and spatial resolution of 2-D echocardiography and strain analysis software have enabled layer-specific strain (LSS) analysis [4]. Several recent studies have reported predominant endocardial strain impairment in patients with subendocardial ischemia, proving the concept that LSS analysis could accurately reflect layer-specific myocardial impairment [5–8]. In the present study, we aimed to elucidate whether LSS could reflect layer-specific myocardial impairment in patients with HCM.

## Materials and Methods

### Study subjects

This study prospectively recruited 67 consecutive patients with HCM according to the definition of the European Society of Cardiology Guidelines on diagnosis and management of HCM [9] at West China Hospital between March 2016 and January 2018. Patients with poor acoustic window (5 patients), LV ejection fraction (LVEF) < 50 % (1 patient), history of septal myectomy (4 patients), atrial fibrillation (1 patient), obstructive coronary artery diseases (> 50 % stenosis on either coronary angiography or computed tomography angiography, 3 patients) or valvular disease (3 patients) were excluded. Ultimately, 50 patients (mean age, 51.2 ± 14.1 years; 50 % women) were enrolled. Thirty age-matched healthy controls with good acoustic windows were also recruited.

### Echocardiography

All echocardiographic data assessments and image acquisitions were performed by an experienced sonographer by using an E9 ultrasonography system (GE Healthcare, Horten, Norway). Routine 2D images were acquired at a frame rate of 40–80 fps. LVEF was measured using the Simpson biplane method. The mitral *E*/*e*′– the ratio of mitral inflow early diastolic velocity (E) to the septal mitral annular spectral tissue Doppler early diastolic velocity (*e’*) – was also obtained in the apical 4-chamber view. Other parameters, such as maximum wall thickness, LV outflow tract gradient, and the phenomenon of systolic anterior motion, were also evaluated in accordance with the recommendations of the American Society of Echocardiography [10].

The 18-segment model, including posterior (basal, mid, and apical), anteroseptal (basal, mid, and apical), septal (basal, mid, and apical), lateral (basal, mid, and apical), inferior (basal, mid, and apical), and anterior (basal, mid, and apical) segments, was used in the segmental analysis. The LV segmental thickness in patients with HCM was measured to identify the hypertrophic (> 12 mm) and normal (≤ 12 mm) segments, and dynamic images were observed. Three consecutive measurements were conducted to find the average value. Images in the apical 4-, 2-, and 3-chamber views from 3 consecutive cycles were stored digitally and analyzed offline.

### LV peak systolic longitudinal strain analyses

**Fig. 1:**
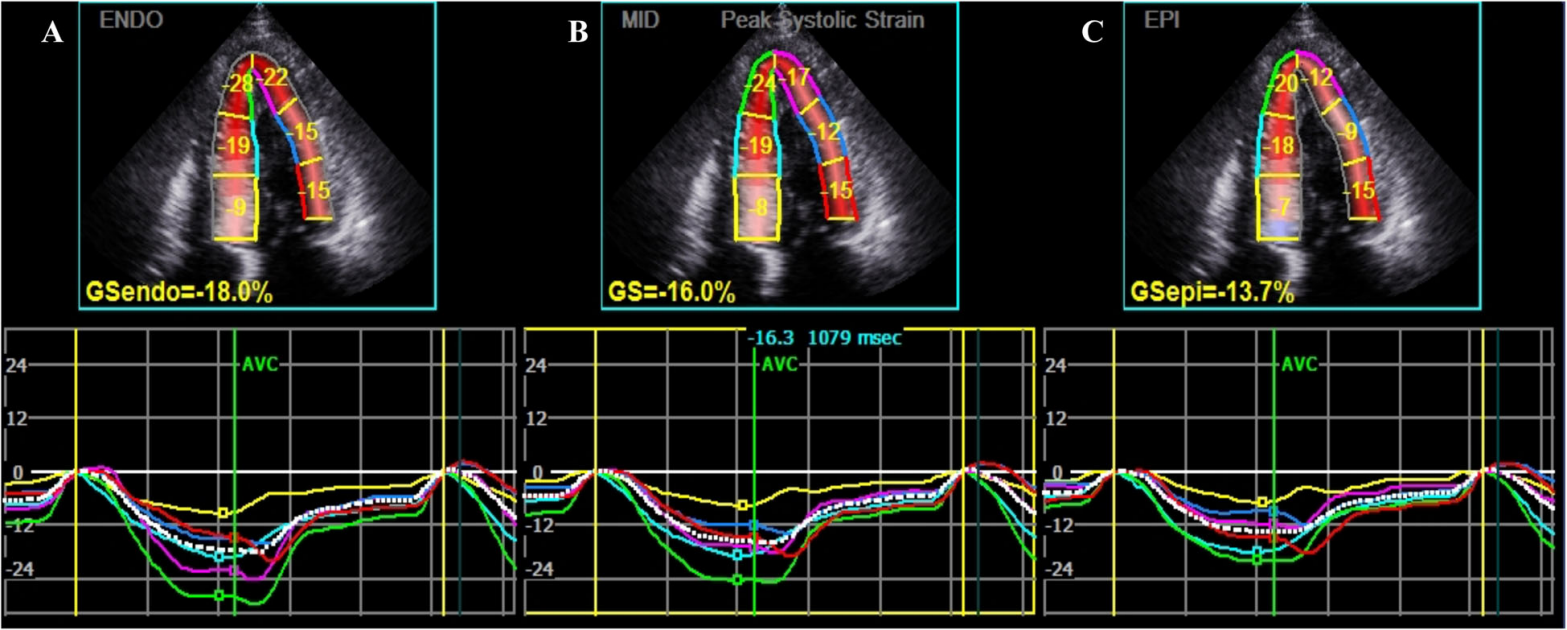
Representative images of two-dimensional (2-D) left ventricular (LV) global layer-specific strain (LSS) analysis in a patient with hypertrophic cardiomyopathy (HCM) from apical 4-chamber views. The 2-D LV global LSS was also measured from apical 2- and 3-chamber views. The analyses were performed using Echo PAC version 201. The quantitative strain measurements of the endocardial (**a**), middle (**b**) and epicardial myocardial layers (**c**) were automatically calculated after the region of interest was manually traced and adjusted

**Fig. 2:**
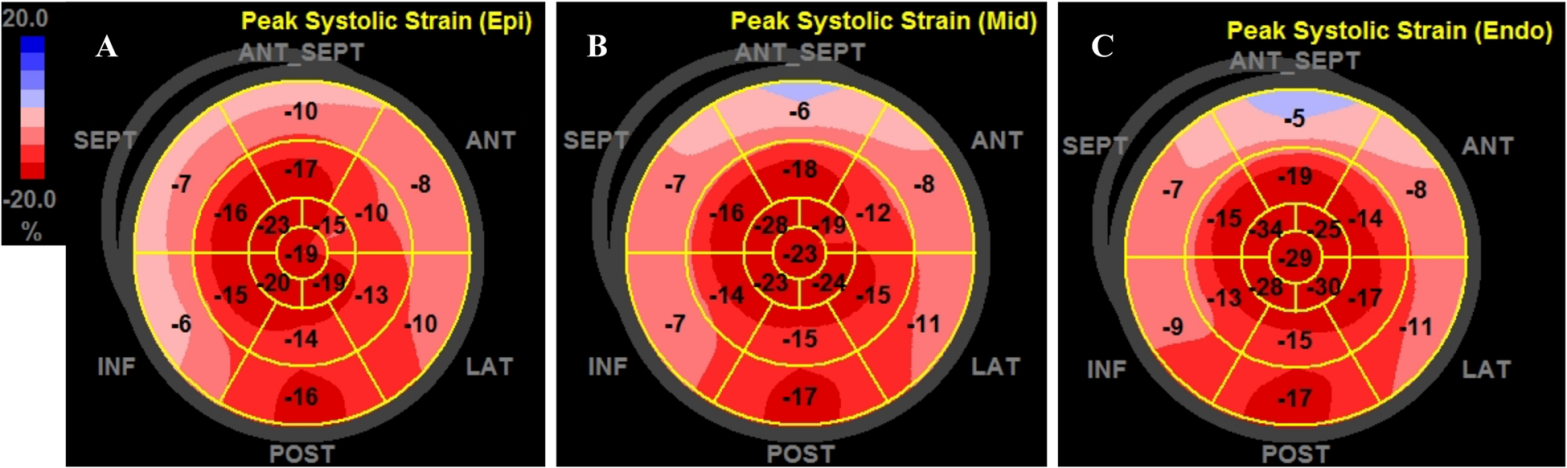
Representative bull’s-eye map of a patient with hypertrophic cardiomyopathy. A multilayer bull’s-eye map, showing the epicardial (**a**), middle (**b**), and endocardial myocardial layers (**c**), was automatically generated after the layer-specific strain was measured in 3 apical views (apical 4-, 2-, and 3-chamber views). An 18-segment model was used in the segmental analysis

The 2-D multilayer global strain in the LV myocardium was analyzed using ECHOPAC version 201 software (GE Healthcare). Aortic valve closing was automatically confirmed by software combined with manual adjustment. The border of the endocardium and the width of the target myocardium were manually traced and adjusted. The poorly traced segments (a total of 14 segments, mostly at the apical levels, of the 50 HCM patients) were also excluded. The software automatically calculated segmental strain of the endocardial, middle, and epicardial myocardial layers (Figs. 1 and 2). The 2-D LV global longitudinal strain was measured from 3 apical views (apical 3-, 2-, and 4-chamber views). The transmural gradient of longitudinal strain (TGLS), a novel parameter defined as the difference between the territorial longitudinal strains of the endocardium and epicardium, was used to reflect endocardium-specific impairment.

### Patients’ follow‐up

Patients were followed up for up to 6 months. Complete clinical follow-up was performed at 6 months, online or phone follow-up was performed at 1 and 3 months. Physicians were free to decide the management strategy during the follow-up period. During the follow-up, 28 HCM patients received adjustment of drug regimens including adding or up-titration of beta blockers (22 patients), adding or up-titration of non-dihydropyridine calcium channel blockers (3 patients) and other changes in regimens (3 patients). Both patients and healthy controls received another echocardiography examination at the end of the follow-up, or before any invasive treatment if invasive strategies including open surgery, septal alcohol ablation or pace-maker implantation were planned.

### Reproducibility of the TGLS-derived parameter

The intra-observer and inter-observer reproducibility of TGLS was measured successively in 10 randomly selected patients with HCM. To test the intra-observer variability, a single observer analyzed the data twice on separate occasions at an interval of 1 month. To test the inter-observer variability, a second observer who was blinded to the first observer’s measurements analyzed the same data.

### Statistical analyses

All statistical analyses were conducted using SPSS version 19.0 (IBM Corporation, Armonk, NY, USA). The Shapiro-Wilk test was used to estimate the normality of the distributions of continuous data. Continuous variables with normal distributions are presented as mean ± standard deviation, whereas those with skewed distributions are expressed as median (interquartile range). Baseline characteristics were compared between the HCM and control groups using independent-samples *t* tests or the Wilcoxon test. Categorical variables were described as counts and percentages, and were compared using the Pearson chi-square test. Comparisons between the different segments, the 3 segmental levels in the controls, and the different subgroups were performed using analysis of variance. Multivariate ordinal logistic regression was used to assess association of different parameters with the grade of severity of abnormal TGLS segments. A receiver operating characteristic (ROC) curve was constructed for the completed model, and a probability threshold was selected for the likelihood of predicting chest pain based on the number of segments with abnormal TGLS. The resulting sensitivity and specificity were also estimated. The intra-observer and inter-observer variability values were analyzed using Bland-Altman bias plots, and by intraclass correlation coefficients (ICC). All tests were 2-sided, and p < 0.05 was considered significant.

## Results

### Study populations

The baseline characteristics and clinical findings of the study participants are listed in Table 1. Most of the HCM patients had septal hypertrophy. The LV mass index was 109.5 ± 35.7 g/m^2^ in HCM group and 68.5 ± 10.4 g/m^2^ in the control group. 64 % of the HCM patients had left ventricular outflow tract (LVOT) obstruction, with a mean resting gradient of 56 mmHg.

**Table 1:** Baseline characteristics of the study population

Variables	HCM (n = 50)	Control (n = 30)	p value
Age, y	51.2 ± 14.1	49.3 ± 10.8	0.968
Female, n (%)	25 (50.0)	14 (46.7)	0.407
Asymmetric septal hypertrophy	48 (96 %)	/	/
Apical hypertrophy	2 (4 %)	/	/
LVOT obstruction	32 (64 %)	/	/
Systolic blood pressure, mmHg	120.3 ± 15.2	119.4 ± 16.9	0.561
Diastolic blood pressure, mmHg	72.8 ± 10.9	71.7 ± 11.5	0.838
Heart rate, bpm	72.9 ± 11.3	76.9 ± 10.4	0.086
Body surface area, m^2^	1.69 ± 0.20	1.63 ± 0.31	0.989
Body mass index, kg/ m^2^	24.8 ± 3.1	23.5 ± 4.3	0.893
Hemoglobin, g/L	135.5 ± 17.4	133.9 ± 19.0	0.865
NT-pro BNP, pg/ml	1059.0 (515.0—2575.0)	/	/
hs-cTnT, ng/L	22.4 (13.4—45.8)	/	/
Creatinine, µmol/L	74.1 ± 17.7	72.4 ± 18.6	0.869
β-blocker, n (%)	38 (76)	/	/
LA diameter, mm	43.8 ± 6.5	31.3 ± 4.7	0.013
LA volume index, ml/ m^2^	34.6 ± 12.8	25.7 ± 7.1	< 0.001
E/e’	21.6 ± 8.0	13.6 ± 11.7	0.008
Maximum wall thickness, mm	21.5 (19.8—25.0)	9.1 ± 1.8	< 0.001
LV mass index, g/m^2^	109.5 ± 35.7	68.5 ± 10.4	< 0.001
Resting LVOT gradient, mmHg	56.0 (9.0—82.0)	7.8 ± 1.6	< 0.001
Systolic anterior motion, n (%)	29 (58)	/	/
Pulmonary artery, mm	22 (21—25)	20.8 ± 2.3	0.003
PASP, mmHg^a^	33 (26—40) (n = 23)	/	/
Cardiac output, L/min	6.57 ± 2.40	5.56 ± 1.72	0.022
LVEF, %	72.7 ± 6.1	64.3 ± 6.4	0.084
Chest pain, n (%)	24 (48)	/	/
Heart failure, n (%)	41 (82)	/	/

NT-pro BNP: N-terminal propeptide of B-type natriuretic peptide; hs-cTnT: high-sensitive cardiac troponin T; LA: left atrial; LVOT: left ventricular outflow tract; E/e’: ratio of early mitral inflow velocity (E) to early diastolic velocity at the septal mitral annulus (e’); PASP: pulmonary arterial systolic pressure; LVEF: left ventricular ejection fraction. The reference values for NT-pro BNP, hs-cTnT, and creatinine are 0–88 pg/ml, 0–14 ng/ml, and 37–110 µmol/L respectively. ^a^: The PASP was estimated using maximal tricuspid regurgitation pressure gradient on the continuous-wave Doppler tracing by the modified Bernoulli equation with the addition of right atrial pressure. Right atrial pressure was estimated at 3 mmHg if the inferior vena cava (IVC) was not dilated (≤ 2.1 cm) and there was a 50 % decrease in the diameter during inspiration; it was estimated at 8 mmHg if the IVC was dilated with normal inspiratory collapse and at 15 mmHg if the IVC was dilated and did not collapse with inspiration

### Echocardiographic findings

**Fig. 3:**
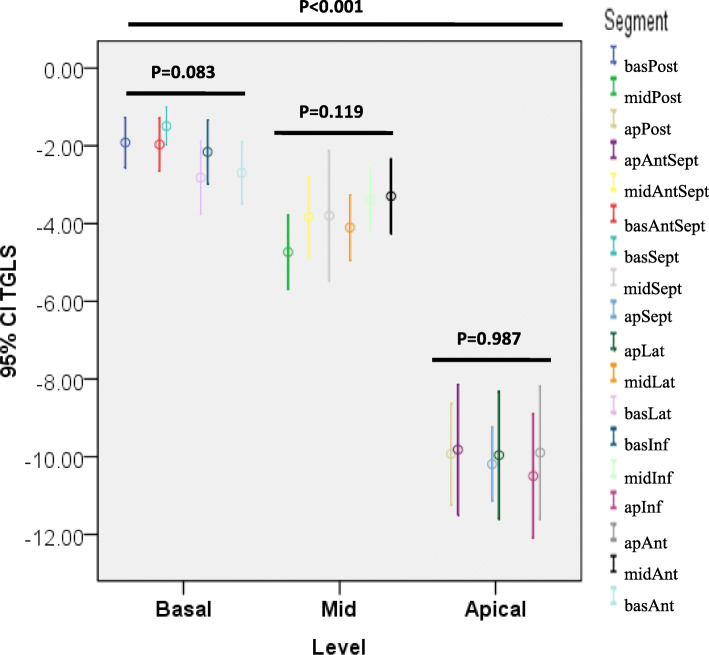
In healthy volunteers, negative transmural gradient of longitudinal strain (TGLS) was observed in almost all segments. The normal range of TGLS greatly varied among the segments. However, the TGLS was relatively consistent within the basal, middle, and apical levels. A significant TGLS from the base toward the apex was observed. bas: basal; ap: apical; Post: posterior; AntSept: anteroseptal; Sept: septal; Lat: lateral; Inf: inferior; Ant: anterior

**Fig. 4:**
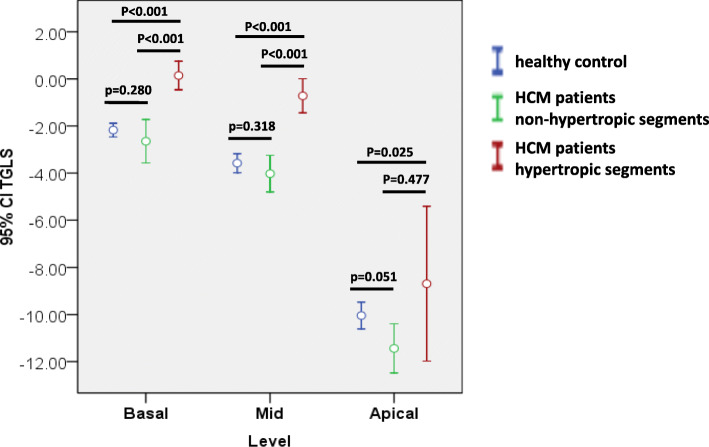
The transmural gradient of longitudinal strain (TGLS) in the non-hypertrophic segments of patients with hypertrophic cardiomyopathy (HCM) was not significantly different from that of healthy controls according to level. However, the TGLS in the hypertrophic segments was significantly higher than that in the non-hypertrophic segments at almost all levels

The layer specific longitudinal strain of healthy volunteers and HCM patients were listed in Table 2. In general, the longitudinal strain of endocardium, mid-myocardium and epicardium were lower in the HCM patients than in the healthy controls. In the healthy volunteers, a negative TGLS (defined as the difference between the territorial longitudinal strains of the endocardium and epicardium) was observed in almost all segments. The normal range of TGLS greatly varied among the different segments (Table 3). A significant TGLS gradient from the base toward the apex was observed (Fig. 3). However, the TGLS was relatively consistent within the basal, middle, and apical levels. In patients with HCM, LV segments were further divided into hypertrophic segments (diastolic thickness of any part within the segment > 12mm) and non-hypertrophic segments (maximum diastolic thickness of the entire segment ≤ 12mm). Among the segments, 55.5 %, 49.8 % and 10.1 % were hypertrophic at the basal, middle, and apical levels, respectively. The TGLS in the non-hypertrophic segments of patients with HCM was not different from that of healthy controls according to level (Table 4; Fig. 4). However, the TGLS in the hypertrophic segments was significantly higher than that in the non-hypertrophic segments at almost all levels.

**Table 2:** Layer-specific strain (%) in healthy controls and HCM patients

Segments	LSS in healthy controls			GLS in healthy patients	LSS in HCM patients			GLS in HCM patients
	endo	mid	epi		endo	mid	epi	
basAntSept	-18.56 ± 3.99	-18.14 ± 3.43	-17.72 ± 3.22	-18.12 ± 3.45	-7.63 ± 5.05*	-7.65 ± 4.66*	-7.71 ± 4.41*	-7.66 ± 4.63*
basSept	-17.82 ± 2.24	-17.74 ± 2.42	-17.67 ± 2.83	-17.75 ± 2.40	-6.25 ± 4.75*	-7.61 ± 4.99*	-8.99 ± 5.29*	-7.62 ± 4.89*
basInf	-20.02 ± 8.39	-19.65 ± 8.34	-19.29 ± 8.44	-19.68 ± 8.32	-11.47 ± 7.30*	-11.66 ± 5.95*	-11.39 ± 5.18*	-11.51 ± 5.99*
basPost	-22.41 ± 4.02	-21.59 ± 3.71	-20.77 ± 3.57	-21.56 ± 3.68	-16.23 ± 9.19*	-15.07 ± 8.09*	-13.62 ± 7.32*	-14.97 ± 8.03*
basLat	-20.60 ± 4.22	-19.45 ± 4.03	-18.29 ± 4.22	-19.40 ± 4.13	-15.50 ± 8.22*	-13.63 ± 6.77*	-11.61 ± 5.92*	-13.58 ± 6.83*
basAnt	-18.24 ± 6.98	-17.42 ± 6.72	-16.60 ± 6.55	-17.45 ± 6.70	-11.46 ± 6.90*	-10.09 ± 6.25*	-8.59 ± 5.83*	-10.05 ± 6.25*
midAntSept	-23.28 ± 5.24	-21.36 ± 4.44	-19.45 ± 3.80	-21.30 ± 4.48	-16.76 ± 7.48*	-14.26 ± 6.14*	-12.22 ± 5.24*	-14.41 ± 6.22*
midSept	-22.29 ± 3.90	-21.13 ± 3.07	-19.98 ± 3.08	-21.11 ± 3.11	-10.37 ± 5.50*	-10.99 ± 4.90*	-11.48 ± 4.60*	-10.95 ± 4.90*
midInf	-22.35 ± 9.17	-21.04 ± 8.74	-19.74 ± 8.43	-21.07 ± 8.78	-11.73 ± 5.57*	-12.38 ± 4.61*	-12.84 ± 4.26*	-12.32 ± 4.64*
midPost	-24.41 ± 4.26	-22.04 ± 3.72	-19.67 ± 3.42	-22.14 ± 3.70	-13.55 ± 7.56*	-12.32 ± 6.30*	-10.95 ± 5.47*	-12.27 ± 6.31*
midLat	-21.69 ± 4.11	-19.69 ± 3.83	-17.70 ± 3.80	-19.65 ± 3.88	-13.96 ± 7.97*	-11.55 ± 6.62*	-9.33 ± 5.73*	-11.61 ± 6.66*
midAnt	-19.98 ± 8.49	-18.59 ± 7.83	-17.21 ± 7.23	-18.54 ± 7.80	-13.48 ± 7.01*	-11.31 ± 6.08*	-9.31 ± 5.45*	-11.36 ± 6.10*
apAntSept	-28.92 ± 7.05	-23.82 ± 5.52	-18.73 ± 4.47	-23.80 ± 5.55	-23.79 ± 13.96	-16.38 ± 9.73*	-11.57 ± 7.27*	-17.25 ± 10.20*
apSept	-30.07 ± 4.79	-24.98 ± 4.26	-19.88 ± 3.94	-24.96 ± 4.24	-23.43 ± 11.56*	-17.04 ± 8.82*	-12.62 ± 7.31*	-17.70 ± 9.01*
apInf	-27.22 ± 14.52	-23.65 ± 11.15	-17.83 ± 9.65	-22.52 ± 12.03	-23.06 ± 11.22	-16.41 ± 7.40*	-11.99 ± 5.61*	-17.15 ± 7.85
apPost	-28.35 ± 5.92	-23.39 ± 4.85	-18.42 ± 4.01	-23.41 ± 4.80	-19.19 ± 12.58*	-13.49 ± 8.89*	-9.45 ± 6.87*	-13.56 ± 9.48*
apLat	-28.00 ± 6.06	-23.02 ± 4.70	-18.04 ± 3.74	-23.12 ± 4.61	-20.31 ± 11.31*	-13.84 ± 8.49*	-9.49 ± 6.80*	-14.55 ± 8.70*
apAnt	-24.18 ± 14.90	-19.59 ± 12.41	-15.00 ± 10.08	-19.57 ± 12.6	-22.15 ± 12.28	-14.95 ± 8.79	-10.07 ± 6.70*	-15.73 ± 9.11
Total	-23.24 ± 8.15	-20.90 ± 6.59	-18.44 ± 5.79	-20.84 ± 6.67	-15.34 ± 10.22*	-12.72 ± 7.37*	-10.73 ± 6.04*	-12.91 ± 7.59*

*LSS* layer-specific strain; *GLS* global longitudinal strain; *HCM* hypertrophic cardiomyopathy; *bas* basal; *ap* apical; *Post* posterior; *AntSept* anteroseptal; *Sept* septal; *Lat* lateral; *Inf* inferior; *Ant* anterior; *endo* endocardial; *mid* middle; *epi* epicardial. * *p* <; 0.05 compared with healthy controls

**Table 3:** Transmural gradient of longitudinal strain (%) in healthy controls and HCM patients

Segments	TGLS in healthy controls		TGLS in HCM patients	
	Mean ± SD	95% CI	Mean ± SD	95% CI
basAntSept	-1.97 ± 1.49	-2.65 to -1.29	0.08 ± 2.20	-0.68 to 0.81
basSept	-1.49±1.06	-1.97 to -1.01	2.73 ± 2.65*	1.89 to 3.59
basInf	-2.16±1.81	-2.98 to -1.33	-0.08 ± 3.93	-1.39 to 1.26
basPost	-1.91 ± 1.42	-2.56 to -1.27	-2.61 ± 4.41	-4.11 to -1.17
basLat	-2.82±2.03	-3.74 to -1.89	-3.90 ± 4.10	-5.31 to -2.64
basAnt	-2.69±1.75	-3.49 to -1.89	-2.87 ± 2.65	-3.77 to -2.05
midAntSept	-3.83±2.27	-4.86 to -2.80	-4.54 ± 3.23	-5.69 to -3.43
midSept	-3.80±3.67	-5.47 to -2.13	1.11 ± 2.58*	0.22 to 1.93
midInf	-3.38±1.79	-4.20 to -2.57	1.11 ± 3.42*	-0.06 to 2.34
midPost	-4.73±2.11	-5.69 to -3.78	-2.60 ± 3.83*	-4.10 to -1.33
midLat	-4.10±1.85	-4.95 to -3.26	-4.64 ± 3.74	-5.84 to -3.45
midAnt	-3.30±2.10	-4.25 to -2.34	-4.17 ± 2.88*	-5.18 to -3.16
apAntSept	-9.82±3.69	-11.49 to -8.14	-12.22 ± 7.62	-15.36 to -9.54
apSept	-10.19±2.08	-11.14 to -9.24	-10.82 ± 6.43	-12.90 to -8.67
apInf	-10.49±3.41	-12.09 to -8.90	-11.06 ± 7.24	-13.69 to -8.46
apPost	-9.93±2.87	-11.23 to -8.62	-9.41 ± 7.22	-12.43 to -6.58
apLat	-9.96±3.60	-11.60 to -8.32	-10.82 ± 6.14	-13.02 to -8.82
apAnt	-9.90±3.64	-11.60 to -8.19	-12.07 ± 6.84	-14.57 to -9.84
Total	-5.33±4.20	-5.76 to -4.91	-4.61 ± 6.72	-5.18 to -4.12

*TGLS* transmural gradient of longitudinal strain; *HCM* hypertrophic cardiomyopathy; *SD* standard deviation; *CI* confidence interval; *bas* basal; *ap* apical; *Post* posterior; *AntSept* anteroseptal; *Sept* septal; *Lat* lateral; *Inf* inferior; *Ant* anterior. ^*^p < 0.05 compared with healthy controls

**Table 4:** TGLS of healthy controls and patients with HCM with normal or hypertrophic segments

Levels	Healthy controls	HCM patients normal segments	HCM patients hypertrophic segments
TGLS basal	−2.17 %±1.66 % (*n* = 180)	−2.65 %±4.44 % (*n* = 133)	0.14 % ± 3.48 %^a, b^ (*n* = 166)
TGLS mid	−3.58 %±2.29 % (*n* = 180)	−4.02 %±4.00 % (*n* = 150)	−0.72 %±3.71 %^a, b^ (*n* = 149)
TGLS apex	−10.04 %±3.20 % (*n* = 180)	−11.44 %±6.65 % (*n* = 259)	−8.69 %±7.96 %^c^ (*n* = 29)

*TGLS* transmural gradient of longitudinal strain; *HCM* hypertrophic cardiomyopathy. ^a^*p* < 0.001, compared with patients with HCM with normal segments. ^b^*p* < 0.001, compared with healthy controls. ^c^*p* < 0.01, compared with healthy controls; no significant difference was observed between the other two pairs.

Abnormal TGLS, defined as a positive TGLS, was observed in 1.1 % (6 of 540) of the normal LV segments in healthy controls. In patients with HCM, 11.3 % (61 of 542) of the non-hypertrophic segments and 45.3 % (156 of 344) of the hypertrophic segments had abnormal TGLS (Table 5.).

**Table 5:** Abnormal TGLS, defined as a positive TGLS, among healthy controls and patients with HCM with normal or hypertrophic segments

Variables	Healthy controls	HCM patients normal segments	HCM patients hypertrophic segments	*p* value
TGLS > 0.0 %, n	6	61	156	/
TGLS ≤ 0.0 %, n	534	481	188	/
Ratio of > 0.0 %	1.1 % (6/540)	11.3 % (61/542)	45.3 % (156/344)	< 0.001

*TGLS* transmural gradient of longitudinal strain; *HCM* hypertrophic cardiomyopathy. The chi-square test was performed for 2-group comparisons among the 3 groups. The *p* values obtained in all the tests were < 0.001. A *p* value of < 0.017 was assumed to be significant.

### Associated with LV hypertrophy and LVOT obstruction

The association of different parameters with the severity of abnormal TGLS segments (defined as the number of abnormal TGLS segments) was assessed in a multivariate ordinal logistic regression. The results demonstrated that LV mass index positively correlated with the severity of abnormal TGLS. In particular, with the increase in LV mass index for every 1 g/m^2^, the OR of abnormal TGLS segments increased by 1.022 times (95 %CI: 1.002–1.043, *p* = 0.032). The presence of LVOT obstruction was also strongly correlated with the severity of abnormal TGLS (OR 5.823, 95 %CI: 1.045–32.464, *p* = 0.044). Other parameters including age, sex, body mass index, width of pulmonary artery, and E/e’ were not significantly associated with the level of abnormal TGLS segments (Table 6).

**Table 6:** Predictors of the severity of abnormal TGLS

	Coefficient B	SE	*P* value	Adjusted OR (95 %CI)
Age	0.043	0.030	0.154	1.044 (0.984–1.107)
Sex/Male	0.174	0.712	0.807	1.190 (0.295–4.808)
BMI, kg/m^2^	0.064	0.124	0.605	1.066 (0.837–1.358)
PA, mm	0.116	0.106	0.275	1.123 (0.912–1.382)
LV mass index, g/m2	0.022	0.010	**0.032**	**1.022 (1.002–1.043)**
LVOTO	1.762	0.877	0.044	5.823 (1.045–32.464)
E/e’	0.049	0.047	0.298	1.050 (0.958–1.151)

*BMI* body mass index; *CI* confidence interval; *E/e’* ratio of early mitral inflow velocity (E) to early diastolic velocity at the septal mitral annulus (e’); *LV* left ventricular; *LVOTO* left ventricular outflow tract obstruction; *OR* odds ratio; *PA* pulmonary artery; *SE* standard error

### Association with symptoms

**Fig. 5:**
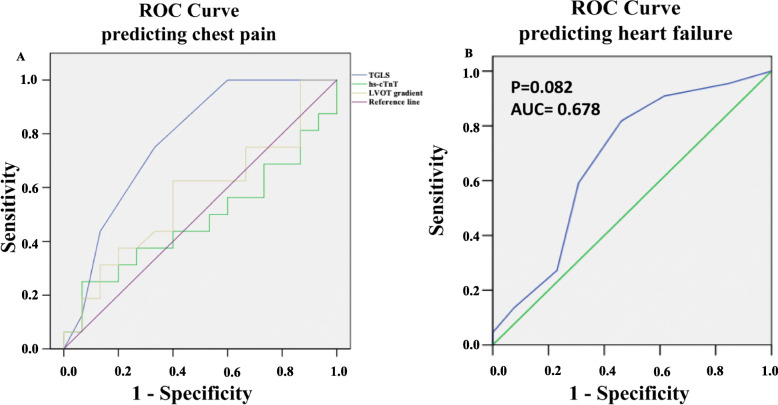
Abnormal transmural gradient of longitudinal strain (TGLS) was significantly associated with chest pain (AUC = 0.805, *p* = 0.002), and a large area of TGLS (> 4 segments) had moderate accuracy for predicting chest pain (sensitivity, 73.3 %; specificity, 70.0 %) in the ROC curve analysis, while hs-cTnT (AUC = 0.479, *p* = 0.843) and LVOT gradient (AUC = 0.564, *p* = 0.528) did not significantly predict chest pain in HCM patients (**a**). Abnormal TGLS was not associated with heart failure (**b**). ROC: receiver operating characteristic; AUC: area under the curve. hs-cTnT: high-sensitive cardiac troponin T; LVOT: left ventricular outflow tract

To further elucidate the clinical significance of abnormal TGLS, patients with HCM were further divided into 2 groups according to symptom. Patients with chest pain generally had more LV segments with abnormal TGLS than those without chest pain (5.4 ± 1.4 vs. 3.5 ± 1.7, p = 0.001). In the ROC curve analysis, a large area of abnormal TGLS (> 4 segments) had moderate accuracy for predicting chest pain (sensitivity, 73.3 %; specificity, 70.0 %) (Fig. 5), while highly-sensitive cardiac troponin T and LVOT gradient did not significantly predict chest pain. Moreover, abnormal TGLS was not associated with heart failure, which was defined as current heart failure according to New York Heart Association class ≥ III, and elevated N-terminal propeptide of B-type natriuretic peptide level.

### TGLS reflect treatment effect

The changes in echocardiographic parameters during follow-up was summarized in Table 7. The patients were further divided into two groups according to whether they received drug adjustment or not. In general, both conventional and strain derived parameters remained similar in healthy controls and patients who did not received drug adjustment. In patients who received adjustment of drug regimens, there is a slight decrease in LVOT pressure gradient, and decreased number of segments with abnormal TGLS.

**Table 7:** TGLS in healthy controls and patients with or without drug adjustment

	Healthy controls (*n* = 30)		Patients without drug adjustment (*n* = 22)		Patients received drug adjustment (*n* = 28)	
	Baseline echo	Follow-up echo	Baseline echo	Follow-up echo	Baseline echo	Follow-up echo
LVEF, %	64.3 ± 6.4	65.6 ± 5.7	70.8 ± 6.1	69.6 ± 7.3	72.7 ± 5.4	73.7 ± 6.5
Resting LVOT gradient, mmHg	7.8 ± 1.6	7.0 ± 2.1	18.2 (6.1—30.3)	18.0 (6.0—30.0)	70.0 (47.0—90.0)	40.0 (17.0—68.0) *
Global LS, %	-20.3 ± 6.84	-22.6 ± 5.49	-12.3 ± 7.87	-12.3 ± 8.79	-13.7 ± 10.10	-14.7 ± 9.70
TGLS basal, %	−2.17 ± 1.66	−2.58 ± 1.03	-1.22 ± 2.10	-1.19 ± 2.70	-1.01 ± 2.14	-1.71 ± 2.98**
TGLS mid, %	−3.58 ± 2.29	−3.27 ± 2.88	-3.28 ± 2.48	-2.98 ± 2.39	-2.72 ± 2.50	-3.31 ± 2.64**
TGLS apex, %	−10.54 ± 3.20	−11.10 ± 4.21	-9.9 ± 3.99	-9.5 ± 3.75	-9.8 ± 4.11	-10.21 ± 3.79
Number of segments with abnormal TGLS	6	8	72	79	145	84***

*LVEF* left ventricular ejection fraction; *LVOT* left ventricular outflow tract; *LS* longitudinal strain; *TGLS* transmural gradient of longitudinal strain. *, **, ***: *p* < 0.05, 0.01, 0.001, compared with baseline data in patients received drug adjustment

### Reproducibility of TGLS

**Fig. 6:**
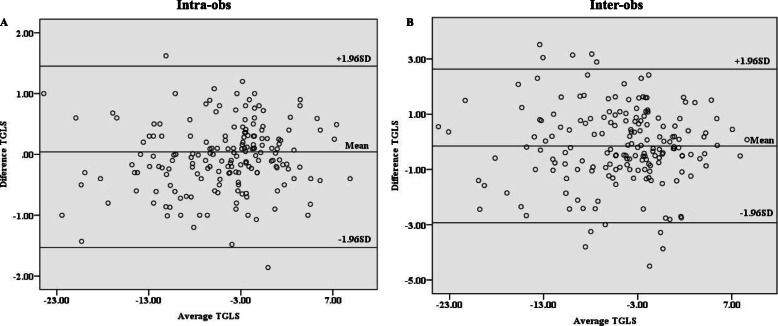
Bland-Altman plots of the intra-observer and inter-observer reproducibility of the transmural gradient of longitudinal strain (TGLS). The limit of agreement was − 1.532–1.449 % for the intra-observer comparison **(a)**, and − 2.929–2.629 % for the inter-observer comparison **(b)**

As shown in Fig. 6, the intra-observer and inter-observer reproducibility of TGLS analysis was relatively good. The ICC for intra-observer and inter-observer analyses were 0.996 and 0.973 respectively.

## Discussion

The principal findings of this study were as follows: (1) TGLS is a reproducible echocardiographic parameter. A negative TGLS was consistently observed in healthy controls. TGLS was significantly decreased from the base toward the apex but was relatively consistent within the basal, middle, and apical levels. (2) In patients with HCM, the hypertrophic LV segments had significantly higher TGLS than the relatively normal segments and healthy controls. (3) The abnormal TGLS in patients with HCM could be associated with subendocardial ischemia and LVOT pressure gradient.

Previous studies have demonstrated that strain imaging was helpful in diagnosis and prognosis prediction in HCM patients [11, 12]. A significant difference between endocardial and epicardial strain has been reported since LSS was developed [4, 13]. Theoretically, strain imaging cannot distinguish the active contraction of the local myocardium and the passive stretching of adjacent layer. This could affect the sensitivity of strain imaging technique for subendocardial dysfunction. In an animal study, multi-layer strain imaging failed to show significant advantage over conventional one-layer strain imaging in detecting subendocardial ischemia [14]. However,the passive stretching effect was outweighed by active contraction in HCM patients since the myocardium was extremely thickened. Recently, Okada et al. [15] investigated HCM patients using LSS. They reported the global longitudinal strain was impaired in patients with HCM and the endocardium was more intensively affected. Interestingly, in this study the transmural gradient of segmental strain had moderate correlation with the segment wall thickness. A very recent large sample echocardiography study also confirmed this correlation [16]. In our study, we found that the transmural gradient of global longitudinal strain was similar in healthy controls and HCM patient. But TGLS was significantly higher in the hypertrophic segments of HCM than in segments with relatively normal thickness or healthy controls.

Although changes in transmural gradient of strain was observed in hypertrophic segments of HCM, its clinical significance has not been clearly elucidated yet. Recent echocardiography studies have reported that endocardial function was more affected in patients with ischemia [5, 6]. A very recent study has further demonstrated that subendocardial strain impairment was a sensitive parameter for early ischemia in dobutamine stressed echocardiography [17]. As a result, we hypothesized that abnormal TGLS was caused by subendocardial ischemia in the extremely hypertrophied myocardium. This hypothesis was supported by the multivariate ordinal logistic regression model demonstrating higher values of the LV mass index significantly and positively increase the likelihood of being in a higher level of abnormal TGLS segments, and by the ROC curve analysis showing large area of abnormal TGLS predicts the onset of chest pain but not heart failure.

Furthermore, a recent study reported improvement of subendocardial strain in patients with hypertrophic obstructive cardiomyopathy after alcohol septal ablation [18], suggesting the abnormality in transmural strain gradient may also be associated with the LVOT obstruction. In our study, nearly two thirds of the HCM patients had significant LVOT obstruction. In a multivariate logistic regression, we found that the severity of abnormal TGLS was associated with not only LV hypertrophy, but also LVOT obstruction. During a 6-month follow-up, 56 % of the HCM patients receiving drug adjustment aimed to lower the LVOT pressure gradient. In these patients, both significant drop in LVOT pressure gradient and decrease in number of segments with abnormal TGLS were observed at the end of the follow-up. Meanwhile, in patients who maintained their regimens, both their LVOT pressure gradient and LSS parameters remained stable during the follow-up. This indicated that LVOT obstruction may contribute to the abnormal subendocardial strain in HCM patients. Interestingly, in two recent studies mainly including non-obstructive HCM patients, the prevalence of positive transmural gradient was very low [15, 16]. These evidences also suggested the LVOT obstruction may contribute to the abnormal transmural strain gradient.

**Fig. 7:**
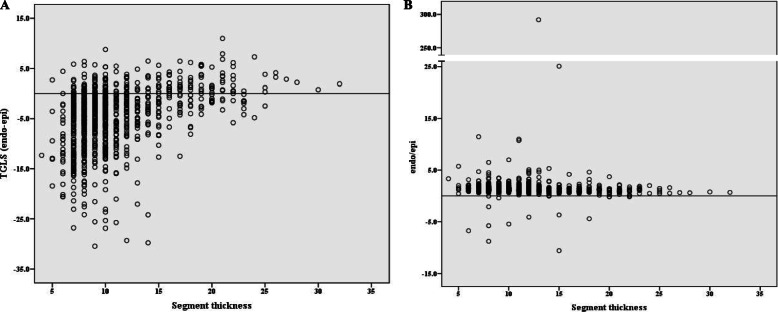
Scatter plot of TGLS **(a)** and the ratio of endocardial strain and epicardial strain **(b)**. Note that there’s an abnormal extreme value in the ratio of endocardial strain and epicardial strain because the epicardial strain was near to zero (-0.02 %)

Another difficulty in interpreting the abnormal transmural strain gradient was the lack of universal measurement and normal reference value. Nagata et al. [19] attempted to determine the normal reference value of LSS in a relatively large number of healthy subjects. In that study, a significant transmural gradient of longitudinal strain from the endocardium toward the epicardium was observed. Nagata et al. suggested an endocardial strain to epicardial strain ratio to reflect this transmural strain gradient. The endocardial strain to epicardial strain ratio was a stable parameter in healthy subjects. However, in patients with abnormal LV deformation, the denominator could be near to zero, which hampered the calculation. Under this circumstance, we believe that the TGLS, defined as the absolute difference in longitudinal strain between the endocardium and the epicardium in a certain LV segment, could be a more reliable parameter for layer-specific LV dysfunction (Fig. 7).

As for the normal reference value, we simply used 0.0 % as the cutoff value for abnormal TGLS in the present study. This cutoff value had relatively high specificity. Healthy controls generally had a negative TGLS. The false-positive rate of TGLS was only 1.1 % in healthy controls. In HCM patients, positive TGLS was found mainly in the hypertrophic segments. However, a significant change of TGLS from the basal toward the apical segments was observed in our study. This trend was also observed by other investigators. In the study by Nagata et al. [19], the average difference between the endocardial and epicardial longitudinal strain was − 2.0 % in the basal segments, -3.4 % in the middle segments, and − 11.4 % in the apical segments. As a result, a level-specific cutoff value might increase the sensitivity. Future studies with larger sample size to determine the individualized TGLS cutoff value for the basal, middle, and apical levels, respectively, would be necessary. This is particularly important for the apical segments, where the normal range of TGLS was significantly lower than in the basal and middle segments. However, in our study, only a small proportion of HCM patients had apical hypertrophy. Furthermore, the relatively poor imaging quality of the apex also hampered accurate strain calculation. Thus, we observed a very wide confidence interval for apical TGLS in HCM patients. Future studies involving more patients with apical HCM might help determine the cutoff value for abnormal TGLS in the apical segments.

### Study limitation

This study has several limitations. Firstly, the major limitation of the present study is the relatively small sample size. Secondly, the possibility that some patients with HCM might also have coronary artery disease but was not totally excluded. Most of the study subjects underwent coronary angiography or coronary computed tomography to rule out potential coronary artery disease. However, in some young and asymptomatic patients, it is unethical to perform invasive coronary angiography or coronary computed tomography. Thirdly, TGLS may be also affected by the presence of microvascular dysfunction in HCM patients. Thus, comparison of echocardiography derived strain with more sensitive perfusion techniques, such as nuclear imaging and CMR, could provide more information. However, only a small portion of our patients received CMR or nuclide myocardial imaging. Thus, comparison of these techniques was not possible in this article. Further studies will be helpful to elucidate the mechanism underlying the layer specific impairment of myocardial strain.

## Conclusions

A novel LSS-derived parameter named TGLS, defined as the difference between the territorial longitudinal strains of the endocardium and epicardium, could reflect the endocardium-specific impairment of regional heart function in patients with HCM.

## Data Availability

The regarding raw data and material of this manuscript can be available through the corresponding author by 69,825,160@qq.com if required.
